# Magneto-Electric Effect on Guided Waves in Functionally Graded Piezoelectric–Piezomagnetic Fan-Shaped Cylindrical Structures

**DOI:** 10.3390/ma11112174

**Published:** 2018-11-02

**Authors:** Bo Zhang, Jiangong Yu, Lahoucine Elmaimouni, Xiaoming Zhang

**Affiliations:** 1 School of Mechanical and Power Engineering, Henan Polytechnic University, Jiaozuo 454003, China; bozhanghpu@163.com (B.Z.); zxmworld11@hpu.edu.cn (X.Z.); 2 LSIE-ERMAM, Faculté Polydisciplinaire d’Ouarzazate, Université Ibn Zohr, 45000 Ouarzazate, Morocco; la_elmaimouni@yahoo.fr

**Keywords:** functionally graded piezoelectric–piezomagnetic material, the Poynting vectors, magneto-electric effect, fan-shaped cross-section, dispersion curves

## Abstract

Functionally graded piezoelectric–piezomagnetic (FGPP) material simultaneously consists of piezomagnetic and piezoelectric phases, which are able to convert energy among mechanical, electric, and magnetic fields. The magneto-electric effect on waves in FGPP fan-shaped cylindrical structures is studied by exploiting the double Legendre orthogonal polynomial method. By means of the Heaviside function, the initial conditions are brought into wave motion equations. Dispersion properties, electric and magnetic potential, and the Poynting vector are calculated. Subsequently, the effect of the graded variation and geometric size on wave characteristics is analyzed. The FGPP fan-shaped cylindrical structures are of complex geometrical shape and material inhomogeneity, so their influences on the magneto-electric effect are the focus of discussion. Results reveal that the cut-off frequencies have a negative relationship with the cross-section area of the structure. The magneto-electric effect could be adjusted via altering the geometric size of the cross-section. These results can be utilized to design and optimize piezoelectric–piezomagnetic fan-shaped transducers.

## 1. Introduction

It is well known that the piezoelectric material has the piezoelectric effect and converse piezoelectric effect, i.e., energy transformation between mechanical and electric fields. Therefore, it has been widely used in various fields, such as semiconductors [1], transducers [2], actuators [3], micromotors [4], and piezoelectric charge-coupled devices [5]. These devices’ performance could be optimized by changing the composition of piezoelectric material [6]. With the development of material science, a class of composite material, the functionally graded piezoelectric–piezomagnetic (FGPP) material, simultaneously consisting of piezomagnetic and piezoelectric phases, was developed, which exhibited a coupled mechanical, magnetic, and electric field. It is able to convert energy among mechanical, electric, and magnetic fields, which is known as the magneto-electric effect [7]. Accordingly, FGPP has promising applications in different areas, such as transducers, sensors, and storage devices [8,9].

To design and optimize the FGPP transducers, wave characteristics in FGPP structures were investigated by utilizing different kinds of models and methods. The propagative behaviors of guided waves in the FGPP plates were investigated by Cao et al. [10], utilizing the power series technique. SH waves, propagating in multi-layered FGPP structures, were studied by Singh and Rokne [11]. By means of the orthogonal polynomial approach, guided wave characteristics in FGPP cylindrical curved plates were studied by Yu et al. [12]. The B–G waves, propagating in an FGPP half-space, were studied by Li et al. [13]. Li and Wei [14] studied the surface wave in FGPP structures, the influences of the initial stresses and the graded function variation on the dispersion curves were analyzed. The longitudinal wave in an FGPP rod was investigated by Xue and Pan [15]. By means of a nonlocal elasticity model, wave propagation in an FGPP nanorod was investigated by Arefi [16]. Narendar [17] studied the wave dispersion in FGPP nonlocal rod. The dispersion properties of circumferential SH wave in different FGPP cylinder shells was studied by Shen et al. [18]. SH wave propagation in FGPP half-space was investigated by Shodja et al. [19]. Hamdi Ezzin et al. investigated waves in piezoelectric/piezomagnetic half-space [20,21] and plates [22] by exploiting the stiffness matrix method. The surface wave characteristics in piezo-composite structures were investigated by Singhal [23] et al., utilizing the WKB (Wentzel-Kramers-Brillouin) method.

The abovementioned waveguides are all of simple geometric cross-sections, i.e., infinite plate, cylinder, and half-space. However, there are also an army of waveguides with complex cross-sections in various applications, such as rectangular bars, rings, and fan-shaped cylindrical structures. The variation of the cross-section is supposed to be one of the efficient ways to govern wave characteristics, such as the wave displacement distribution and cut-off frequencies. Moreover, the performance of the FGPP devices is closely tied with wave characteristics, so their performance can be improved by changing geometry size. Accordingly, waves in waveguides with complex cross-sections have become a hot topic in recent years. By using the double Legendre orthogonal polynomial method, wave characteristics in FGPP rectangular bars were investigated by Zhang et al. [24]. Zhou et al. [25] studied elastic waves propagating in multiferroic cylinders of fan-shaped cross-section. Guided waves propagating in piezoceramic fan-shaped cylinders were studied by Puzyrev [26,27], and the effect of the variation in angular measure on wave characteristics was analyzed. Storozhev [28] studied elastic waves propagating in layered piezoelectric cylinders of a fan-shaped notch. For the fan-shaped cylindrical structures in Ref. [25,26,27,28], the amplitudes of displacements are assumed to be a function with respect to the variable *r*, i.e., when the angle *θ* is defined, and the amplitudes of displacements are just unknown with respect to the variable *r*. Recently, Zhang et al. [29] proposed a new 2-D model, in which the amplitudes of displacements are completely unknown with respect to the variables *r* and *θ*, to investigate the complex guided waves in fan-shaped cylindrical structures. To our best knowledge, rare references about the waves in 2-D FGPP fan-shaped cylindrical structures are available. Besides, the numerical methods, such as the finite element method [30] and the semi-analytical finite element method [31], are usually time-consuming for investigating waves propagating in 2-D structures. Therefore, an analytic method, i.e., the double orthogonal polynomial method, is exploited to investigate waves in 2-D structures, which has two features: (1) each independent variable can be expanded into an proper series of Legendre orthogonal polynomials, and the governing differential equations would be transformed into a matrix eigenvalue problem, and the amplitudes of displacements can be obtained according to the eigenvectors. (2) It directly incorporates the boundary conditions into the dynamic motion equations via assuming position-dependent material constants, which simplifies the solution procedure.

To design and optimize the FGPP cylindrical transducers, the magneto-electric effect on waves in FGPP fan-shaped cylindrical structures is studied via exploiting the double Legendre orthogonal polynomial method. The initial conditions, i.e., traction-free and open-circuit, are given.

## 2. Mathematics and Formulation

**Figure 1:**
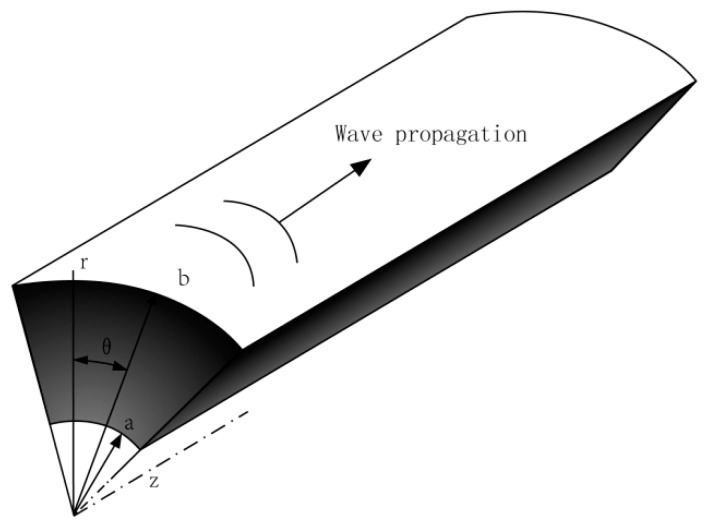
Schematic drawing of a functionally graded piezoelectric–piezomagnetic (FGPP) fan-shaped cylindrical structure.

Consider a functionally graded piezoelectric-piezomagnetic (FGPP) fan-shaped cylindrical structure in the cylindrical coordinate (*θ*, *z*, *r*), as illustrated in the Figure 1. The polarization and gradient directions are both assumed to be along the radial direction. The radius–thickness ratio is represented as *η*, *η* = *b*/(*b* − *a*), where *a* and *b* are inner and outer radius, respectively.

For the FGPP cylindrical structures, the gradient direction is along the *r* direction. Accordingly, material parameters are compactly expressed as the following form, via the least square method.

(1) $$\begin{array}{c} C_{ijkl}(r)=\sum_{l=0}^{L}C_{ijkl}^{\left(l\right)}{\left(\frac{r}{d}\right)}^{l}I(r,\theta ),\rho (r)=\sum_{l=0}^{L}\rho^{\left(l\right)}{\left(\frac{r}{d}\right)}^{l}I(r,\theta ),e_{jkl}(r)=\sum_{l=0}^{L}e_{jkl}^{\left(l\right)}{\left(\frac{r}{d}\right)}^{l}I(r,\theta ),\\ \in_{jk}(r)=\sum_{l=0}^{L}\in_{jk}^{\left(l\right)}{\left(\frac{r}{d}\right)}^{l}I(r,\theta ),q_{jkl}(r)=\sum_{l=0}^{L}q_{jkl}^{\left(l\right)}{\left(\frac{r}{d}\right)}^{l}I(r,\theta ),\\ g_{jk}(r)=\sum_{l=0}^{L}g_{jk}^{\left(l\right)}{\left(\frac{r}{d}\right)}^{l}I(r,\theta ),\mu_{jk}(r)=\sum_{l=0}^{L}\mu_{jk}^{\left(l\right)}{\left(\frac{r}{d}\right)}^{l}I(r,\theta ). \end{array}$$

Here, the function *I*(*r*, *θ*) [32] is introduced into Equation (1), to automatically satisfy the traction-free and open-circuit boundary conditions, which can be denoted as follows:

(2) $$\left\{ \begin{array}{l} T_{rr}=T_{r\theta }=T_{rz}=D_{r}=B_{r}=0,\;r=a\;and\;r=b\\ T_{\theta \theta }=T_{r\theta }=T_{\theta z}=D_{\theta }=B_{\theta }=0,\;\theta =0\;and\;\theta =\alpha \end{array}\right..$$

The harmonic wave solutions are assumed as

(3) $$\begin{array}{l} u_{r}(r,\theta ,z,t)=\exp(ikz-i\omega t)U(r,\theta ),\\ u_{\theta }(r,\theta ,z,t)=\exp(ikz-i\omega t)V(r,\theta ),\\ u_{z}(r,\theta ,z,t)=\exp(ikz-i\omega t)W(r,\theta ),\\ \Phi (r,\theta ,z,t)=\exp(ikz-i\omega t)X(r,\theta ),\\ \Psi (r,\theta ,z,t)=\exp(ikz-i\omega t)Y(r,\theta ), \end{array}$$

where *U*(*r*, *θ*), *V*(*r*, *θ*), and *W*(*r*, *θ*) denote the amplitude in the *r*, *θ*, and *z* directions, respectively. *X*(*r*, *θ*) and *Y*(*r*, *θ*) are the amplitude of electric and magnetic potential, respectively. *k* represents the wave number, and *ω* is the angular frequency.

Subsequently, substituting the Equations (1)–(3), the constitutive equations, and the relation of extend strain-displacement into the dynamic equation [12,33], the following governing differential equations are obtained.

(4a) $$\begin{array}{l} {\left(r/d\right)}^{l}\{I(r,\theta )[C_{33}^{\left(l\right)}r^{2}U{,}_{rr}+C_{55}^{\left(l\right)}U{,}_{\theta \theta }+C_{33}^{\left(l\right)}(l+1)rU{,}_{r}-(C_{44}^{\left(l\right)}k^{2}r^{2}+C_{11}^{\left(l\right)})U+C_{13}^{\left(l\right)}lU+\\ (C_{13}^{\left(l\right)}+C_{55}^{\left(l\right)})rV{,}_{r\theta }-(C_{11}^{\left(l\right)}+C_{55}^{\left(l\right)})V{,}_{\theta }+C_{13}^{\left(l\right)}lV{,}_{\theta }+(C_{23}^{\left(l\right)}+C_{44}^{\left(l\right)})ikr^{2}W{,}_{r}+(C_{23}^{\left(l\right)}-C_{12}^{\left(l\right)})ikrW\\ +C_{23}^{\left(l\right)}likrW-e_{24}^{\left(l\right)}k^{2}r^{2}X+(e_{33}^{\left(l\right)}-e_{31}^{\left(l\right)})rX{,}_{r}+e_{33}^{\left(l\right)}lrX{,}_{r}+e_{33}^{\left(l\right)}r^{2}X{,}_{rr}+e_{15}^{\left(l\right)}X{,}_{\theta \theta }\\ -q_{24}^{\left(l\right)}k^{2}r^{2}Y+(q_{33}^{\left(l\right)}-q_{31}^{\left(l\right)})rY{,}_{r}+q_{33}^{\left(l\right)}lrY{,}_{r}+q_{33}^{\left(l\right)}r^{2}Y{,}_{rr}+q_{15}^{\left(l\right)}Y{,}_{\theta \theta }]\\ +I(r,\theta ){,}_{r}(C_{33}^{\left(l\right)}r^{2}U{,}_{r}+C_{13}^{\left(l\right)}rU+C_{13}^{\left(l\right)}rV{,}_{\theta }+C_{23}^{\left(l\right)}ikr^{2}W+e_{33}^{\left(l\right)}r^{2}X{,}_{r}+q_{33}^{\left(l\right)}r^{2}Y{,}_{r})\\ +I(r,\theta ){,}_{\theta }(C_{55}^{\left(l\right)}U{,}_{\theta }+C_{55}^{\left(l\right)}rV{,}_{r}-C_{55}^{\left(l\right)}V+e_{15}^{\left(l\right)}X{,}_{\theta }+q_{15}^{\left(l\right)}Y{,}_{\theta })\}=-{\left(r/d\right)}^{l}\rho^{\left(l\right)}\omega^{2}r^{2}U \end{array}$$

(4b) $$\begin{array}{l} {\left(r/d\right)}^{l}\{I(r,\theta )[(C_{55}^{\left(l\right)}+C_{13}^{\left(l\right)})rU{,}_{r\theta }+(C_{55}^{\left(l\right)}+C_{11}^{\left(l\right)})U{,}_{\theta }+C_{55}^{\left(l\right)}lU{,}_{\theta }+C_{55}^{\left(l\right)}r^{2}V{,}_{rr}+C_{11}^{\left(l\right)}V{,}_{\theta \theta }+C_{55}^{\left(l\right)}(l+1)rV{,}_{r}\\ -(C_{66}^{\left(l\right)}k^{2}r^{2}+C_{55}^{\left(l\right)})V-C_{55}^{\left(l\right)}lV+(C_{12}^{\left(l\right)}+C_{66}^{\left(l\right)})ikrW{,}_{\theta }+e_{15}^{\left(l\right)}(l+1)X{,}_{\theta }+(e_{15}^{\left(l\right)}+e_{31}^{\left(l\right)})rX{,}_{r\theta }+q_{15}^{\left(l\right)}(l+1)Y{,}_{\theta }\\ +(q_{15}^{\left(l\right)}+q_{31}^{\left(l\right)})rY{,}_{r\theta }]+I(r,\theta ){,}_{r}(C_{55}^{\left(l\right)}rU{,}_{\theta }+C_{55}^{\left(l\right)}r^{2}V{,}_{r}-C_{55}^{\left(l\right)}rV+e_{15}^{\left(l\right)}rX{,}_{\theta }+q_{15}^{\left(l\right)}rY{,}_{\theta })\\ +I(r,\theta ){,}_{\theta }(C_{11}^{\left(l\right)}V{,}_{\theta }+C_{12}^{\left(l\right)}ikrW+C_{11}^{\left(l\right)}U+C_{13}^{\left(l\right)}rU{,}_{r}+e_{31}^{\left(l\right)}rX{,}_{r}+q_{31}^{\left(l\right)}rY{,}_{r})\}=-{\left(r/d\right)}^{l}\rho^{\left(l\right)}\omega^{2}r^{2}V \end{array}$$

(4c) $$\begin{array}{l} {\left(r/d\right)}^{l}\{I(r,\theta )[(C_{44}^{\left(l\right)}+C_{23}^{\left(l\right)})ikr^{2}U{,}_{r}+(C_{44}^{\left(l\right)}+C_{12}^{\left(l\right)})ikrU+C_{44}^{\left(l\right)}likrU+(C_{12}^{\left(l\right)}+C_{66}^{\left(l\right)})ikrV{,}_{\theta }\\ +C_{44}^{\left(l\right)}r^{2}W{,}_{rr}+C_{66}^{\left(l\right)}W{,}_{\theta \theta }+C_{44}^{\left(l\right)}(l+1)rW{,}_{r}-C_{22}^{\left(l\right)}k^{2}r^{2}W+e_{24}^{\left(l\right)}(l+1)ikrX+(e_{24}^{\left(l\right)}+e_{32}^{\left(l\right)})ikr^{2}X{,}_{r}\\ +q_{24}^{\left(l\right)}(l+1)ikrY+(q_{24}^{\left(l\right)}+q_{32}^{\left(l\right)})ikr^{2}Y{,}_{r}]+I(r,\theta ){,}_{r}(C_{44}^{\left(l\right)}ikr^{2}U+C_{44}^{\left(l\right)}r^{2}W{,}_{r}+e_{24}^{\left(l\right)}ikr^{2}X\\ +q_{24}^{\left(l\right)}ikr^{2}Y)+I(r,\theta ){,}_{\theta }(C_{66}^{\left(l\right)}ikrV+C_{66}^{\left(l\right)}W{,}_{\theta })\}=-{\left(r/d\right)}^{l}\rho^{\left(l\right)}\omega^{2}r^{2}W \end{array}$$

(4d) $$\begin{array}{l} {\left(r/d\right)}^{l}\{I(r,\theta )[-e_{24}^{\left(l\right)}k^{2}r^{2}U+e_{31}^{\left(l\right)}lU+(e_{31}^{\left(l\right)}+e_{33}^{\left(l\right)})rU{,}_{r}+e_{33}^{\left(l\right)}lrU{,}_{r}+e_{33}^{\left(l\right)}r^{2}U{,}_{rr}+e_{15}^{\left(l\right)}U{,}_{\theta \theta }-e_{15}^{\left(l\right)}V{,}_{\theta }+e_{31}^{\left(l\right)}lV{,}_{\theta }\\ +(e_{31}^{\left(l\right)}+e_{15}^{\left(l\right)})rV{,}_{r\theta }+e_{32}^{\left(l\right)}(l+1)ikrW+(e_{32}^{\left(l\right)}+e_{24}^{\left(l\right)})ikr^{2}W{,}_{r}+\in_{22}^{\left(l\right)}k^{2}r^{2}X-\in_{33}^{\left(l\right)}(l+1)rX{,}_{r}-\in_{33}^{\left(l\right)}r^{2}X{,}_{rr}\\ -\in_{11}^{\left(l\right)}X{,}_{\theta \theta }+g_{22}^{\left(l\right)}k^{2}r^{2}Y-g_{33}^{\left(l\right)}(l+1)rY{,}_{r}-g_{33}^{\left(l\right)}r^{2}Y{,}_{rr}-g_{11}^{\left(l\right)}Y{,}_{\theta \theta }]+I(r,\theta ){,}_{r}(e_{31}^{\left(l\right)}rU+e_{33}^{\left(l\right)}r^{2}U{,}_{r}+e_{31}^{\left(l\right)}rV{,}_{\theta }\\ +e_{32}^{\left(l\right)}ikr^{2}W-\in_{33}^{\left(l\right)}r^{2}X{,}_{r}-g_{33}^{\left(l\right)}r^{2}Y{,}_{r})+I(r,\theta ){,}_{\theta }(e_{15}^{\left(l\right)}U{,}_{\theta }-e_{15}^{\left(l\right)}V+e_{15}^{\left(l\right)}rV{,}_{r}-\in_{11}^{\left(l\right)}X{,}_{\theta }-g_{11}^{\left(l\right)}Y{,}_{\theta })\}=0 \end{array}$$

(4e) $$\begin{array}{l} {\left(r/d\right)}^{l}\{I(r,\theta )[-q_{24}^{\left(l\right)}k^{2}r^{2}U+q_{31}^{\left(l\right)}lU+(q_{31}^{\left(l\right)}+q_{33}^{\left(l\right)})rU{,}_{r}+q_{33}^{\left(l\right)}lrU{,}_{r}+q_{33}^{\left(l\right)}r^{2}U{,}_{rr}+q_{15}^{\left(l\right)}U{,}_{\theta \theta }-q_{15}^{\left(l\right)}V{,}_{\theta }+q_{31}^{\left(l\right)}lV{,}_{\theta }\\ +(q_{31}^{\left(l\right)}+q_{15}^{\left(l\right)})rV{,}_{r\theta }+q_{32}^{\left(l\right)}(l+1)ikrW+(q_{32}^{\left(l\right)}+q_{24}^{\left(l\right)})ikr^{2}W{,}_{r}+g_{22}^{\left(l\right)}k^{2}r^{2}X-g_{33}^{\left(l\right)}(l+1)rX{,}_{r}-g_{33}^{\left(l\right)}r^{2}X{,}_{rr}\\ -g_{11}^{\left(l\right)}X{,}_{\theta \theta }+\mu_{22}^{\left(l\right)}k^{2}r^{2}Y-\mu_{33}^{\left(l\right)}(l+1)rY{,}_{r}-\mu_{33}^{\left(l\right)}r^{2}Y{,}_{rr}-\mu_{11}^{\left(l\right)}Y{,}_{\theta \theta }]+I(r,\theta ){,}_{r}(q_{31}^{\left(l\right)}rU+q_{33}^{\left(l\right)}r^{2}U{,}_{r}+q_{31}^{\left(l\right)}rV{,}_{\theta }\\ +q_{32}^{\left(l\right)}ikr^{2}W-g_{33}^{\left(l\right)}r^{2}X{,}_{r}-\mu_{33}^{\left(l\right)}r^{2}Y{,}_{r})+I(r,\theta ){,}_{\theta }(q_{15}^{\left(l\right)}U{,}_{\theta }-q_{15}^{\left(l\right)}V+q_{15}^{\left(l\right)}rV{,}_{r}-g_{11}^{\left(l\right)}X{,}_{\theta }-u_{11}^{\left(l\right)}Y{,}_{\theta })\}=0 \end{array}$$

where the subscript comma denotes partial derivative.

Subsequently, *U*(*r*, *θ*), *V*(*r*, *θ*), *W*(*r*, *θ*), *X*(*r*, *θ*), and *Y*(*r*, *θ*) are expanded into the double Legendre orthogonal polynomial series.

(5) $$\begin{array}{c} U(r,\theta )=\sum_{m,j=0}^{\infty }p_{m,j}^{1}Q_{m}(r)Q_{j}(\theta ),\;V(r,\theta )=\sum_{m,j=0}^{\infty }p_{m,j}^{2}Q_{m}(r)Q_{j}(\theta ),\;W(r,\theta )=\sum_{m,j=0}^{\infty }p_{m,j}^{3}Q_{m}(r)Q_{j}(\theta ),\\ X(r,\theta )=\sum_{m,j=0}^{\infty }p_{m,j}^{4}Q_{m}(r)Q_{j}(\theta ),\;Y(r,\theta )=\sum_{m,j=0}^{\infty }p_{m,j}^{5}Q_{m}(r)Q_{j}(\theta ), \end{array}$$

where $p_{m,j}^{i}(i=1,2,3,4,5)$ denote expansion coefficients and

(6) $$Q_{m}(r)=\sqrt{\frac{2m+1}{d}}P_{m}\left(\frac{2r-d}{d}\right),\;Q_{j}(\theta )=\sqrt{\frac{2j+1}{\beta }}P_{j}\left(\frac{2\theta -\beta }{\beta }\right),$$

where $P_{m}$ and $P_{j}$ represent the Legendre polynomials with the order *m* and *j*, respectively. *m* and *j* are evaluated from 0 to ∞ in theory. However, as a matter of fact, the Equation (5) are convergent at some finite values *m* = *M* and *j* = *J*.

Multiply Equations (4a)–(4e) by $Q_{n}(r)\times Q_{p}(\theta )$, with *n* running from 0 to *M* and *p* running from 0 to *J*, respectively. Subsequently, integrating over *r* from *a* to *b*, and over *θ* from 0 to *β*. Then, we can obtain the following system by utilizing the orthonormality:

(7a) $${}^{l}A_{11}^{n,p,m,j}p_{m,j}^{1}+{}^{l}A_{12}^{n,p,m,j}p_{m,j}^{2}+{}^{l}A_{13}^{n,p,m,j}p_{m,j}^{3}+{}^{l}A_{14}^{n,p,m,j}p_{m,j}^{4}+{}^{l}A_{11}^{n,p,m,j}p_{m,j}^{5}=-\omega^{2}{}^{l}M_{n,p,m,j}p_{m,j}^{1},$$

(7b) $${}^{l}A_{21}^{n,p,m,j}p_{m,j}^{1}+{}^{l}A_{22}^{n,p,m,j}p_{m,j}^{2}+{}^{l}A_{23}^{n,p,m,j}p_{m,j}^{3}+{}^{l}A_{24}^{n,p,m,j}p_{m,j}^{4}+{}^{l}A_{25}^{n,p,m,j}p_{m,j}^{5}=-\omega^{2}{}^{l}M_{n,p,m,j}p_{m,j}^{2},$$

(7c) $${}^{l}A_{31}^{n,p,m,j}p_{m,j}^{1}+{}^{l}A_{32}^{n,p,m,j}p_{m,j}^{2}+{}^{l}A_{33}^{n,p,m,j}p_{m,j}^{3}+{}^{l}A_{34}^{n,p,m,j}p_{m,j}^{4}+{}^{l}A_{35}^{n,p,m,j}p_{m,j}^{5}=-\omega^{2}{}^{l}M_{n,p,m,j}p_{m,j}^{3},$$

(7d) $${}^{l}A_{41}^{n,p,m,j}p_{m,j}^{1}+{}^{l}A_{42}^{n,p,m,j}p_{m,j}^{2}+{}^{l}A_{43}^{n,p,m,j}p_{m,j}^{3}+{}^{l}A_{44}^{n,p,m,j}p_{m,j}^{4}+{}^{l}A_{45}^{n,p,m,j}p_{m,j}^{5}=0,$$

(7e) $${}^{l}A_{51}^{n,p,m,j}p_{m,j}^{1}+{}^{l}A_{52}^{n,p,m,j}p_{m,j}^{2}+{}^{l}A_{53}^{n,p,m,j}p_{m,j}^{3}+{}^{l}A_{54}^{n,p,m,j}p_{m,j}^{4}+{}^{l}A_{55}^{n,p,m,j}p_{m,j}^{5}=0,$$

where ${}^{l}M_{n,p,m,j}$ and ${}^{l}A_{\alpha \gamma }^{n,p,m,j}\;(\alpha ,\gamma =1,2,3,4,5)$, which can be calculated based on Equation (6), are the elements of the non-symmetric matrices.

Equation (7) can be transformed as the following matrix system, and the detailed transformation process is given in the Appendix A.

(8) $$\left[\begin{array}{ccc} {}^{l}{\bar{A}}_{11}^{n,p,m,j} & {}^{l}{\bar{A}}_{12}^{n,p,m,j} & {}^{l}{\bar{A}}_{13}^{n,p,m,j}\\ {}^{l}{\bar{A}}_{21}^{n,p,m,j} & {}^{l}{\bar{A}}_{22}^{n,p,m,j} & {}^{l}{\bar{A}}_{22}^{n,p,m,j}\\ {}^{l}{\bar{A}}_{31}^{n,p,m,j} & {}^{l}{\bar{A}}_{32}^{n,p,m,j} & {}^{l}{\bar{A}}_{33}^{n,p,m,j} \end{array}\right]\left\{\begin{array}{c} p_{m,j}^{1}\\ p_{m,j}^{2}\\ p_{m,j}^{3} \end{array}\right\}=-\omega^{2}\left[\begin{array}{ccc} {}^{l}M_{n,p,m,j} & 0 & 0\\ 0 & {}^{l}M_{n,p,m,j} & 0\\ 0 & 0 & {}^{l}M_{n,p,m,j} \end{array}\right]\left\{\begin{array}{c} p_{m,j}^{1}\\ p_{m,j}^{2}\\ p_{m,j}^{3} \end{array}\right\}$$

where the dimensions of matrices ${}^{l}{\bar{A}}_{\alpha \gamma }^{n,p,m,j}\;(\alpha ,\gamma =1,2,3)$ and ${}^{l}M_{n,p,m,j}$ are all (*M* + 1) × (*J* + 1).

Consequently, Equation (8) is an eigenvalue problem about angular frequency *ω*. The profiles of displacement components are calculated according to the eigenvectors. At last, the phase velocity is obtained via the equation *V_ph_*= *ω*/*k.*

## 3. Numerical Results

In this section, the equivalent parameters of the FGPP cylindrical structures are calculated utilizing the Voigt-type model [29].

According to the abovementioned equations, the computer programs in the light of the double orthogonal polynomial method are written using the software “Mathematica” to calculate the dispersion curves and the Poynting vectors.

In the present paper, such a kind of FGPP fan-shaped cylindrical structure is considered: the inner layer material is CoFe_2_O_4_, and the outer layer material is BaTiO_3_. Their material constants are listed in the Table 1 in the reference [34].

### 3.1. Comparison with Rectangular Bar

**Figure 2:**
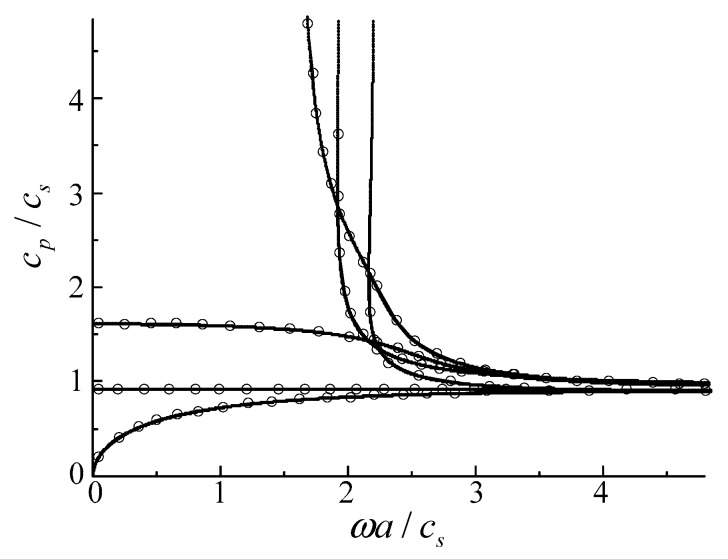
Phase velocity curves for the square bars: dotted lines—results from the reference [35], lines—the results of the present method.

As far as we know, rare references for the two-dimensional FGPP fan-shaped cylindrical structures are available. Consequently, we simplify the model as a purely elastic structure, and assume its geometric size is *a* = (10^−4^ − 1) mm, *b* = 10^4^ mm, and *β* = 10^−4^ rad, respectively, to compare with a square steel bar using the two dimensional Rayleigh–Ritz method [35]. Its cross-section area is 1 mm^2^. Their phase velocity curves are shown in the Figure 2, where the lines are the authors’ results, and the dotted lines are results from reference [35]. *c_p_* represents the phase velocity, and *c_s_* represents the shear velocity. These results of two methods are overlapped completely. Accordingly, the correctness of the present method is confirmed.

**Figure 3:**
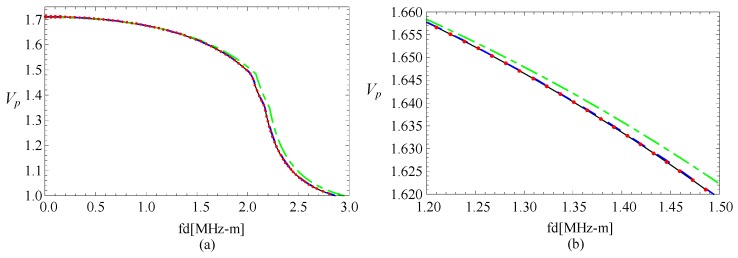
Comparison of dispersion curves for the fan-shaped cylindrical structure cross-section with different *η* and square bar; (**b**) is the enlarged drawing of (**a**).

Subsequently, a comparison with an FGPP square bar from reference [24] is made, and the influence of the radius-thickness ratio is analyzed. To ensure the area of cross-section has very little difference, their geometric parameters are *η* = 10 and *β* = 1/9.5 rad, *η* = 100 and *β* = 1/99.5 rad, and *η* = 1000 and *β* = 1/999.5 rad, respectively. Their thicknesses are both 1 mm. Their phase velocity curves for the fourth mode are illustrated in the Figure 3. The cases of the other modes are similar. *Vp* is a dimensionless parameter (*Vp* = (c_p_*d)/c_*s*1_, where *c_p_* is the phase velocity value, and c_*s*1_ is the shear velocity value of CoFe_2_O_4_). Here, Figure 3b is the partial enlarged drawing of Figure 3a. We can note from these figures that the phase velocity for the fan-shaped cylindrical structures is getting closer to that of rectangular bar as the radius-thickness ratio increases. Moreover, the dotted line and the line are overlapped completely as the radius-thickness ratio *η* = 1000.

### 3.2. Convergence Confirmation

To confirm the convergence of the present method, the phase velocities of a linear FGPP fan-shaped cylindrical structure (*β* = π/6 and *η* = 2) with various *M* and *J* are calculated. Their phase velocity values are listed in Table 1 at *kd* = 1.01.

Here, as *M* or *J* varies, the relative error is

(9) $$\Delta =(c_{\left(M+1)*J\mathrm{orM}*(J+1\right)}-c_{M*J})/c_{M*J}\leq \frac{1}{1000}.$$

It is convergent in the present paper. *c* represents the phase velocity. We can note that the first two modes are convergent as *M* = *J* = 5, and the first three modes are convergent as *M* = 6 and *J* = 5.

### 3.3. The Magneto-Electric Effect

**Figure 4:**
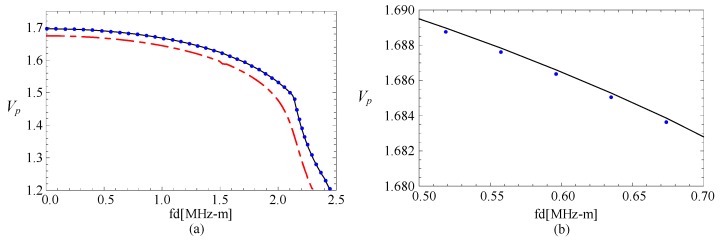
The phase velocity curves: the line—FGPP structure; dotted line—piezoelectric structure; dashed line—piezomagnetic structure; (**b**) is the enlarged drawing of (**a**).

**Figure 5:**
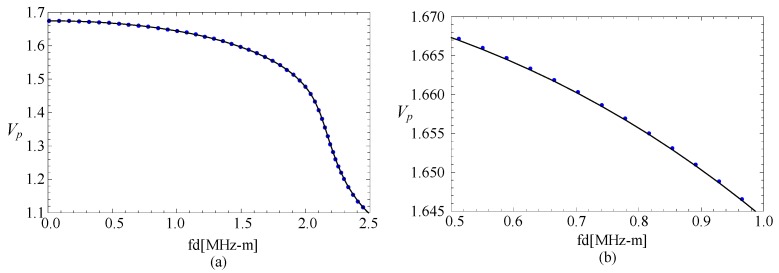
The phase velocity curves, the line—elastic structure, dotted line—piezomagnetic structure; (**b**) is the enlarged drawing of (**a**).

The phase velocity curves of FGPP fan-shaped cylindrical structure, corresponding graded piezoelectric structure, and graded piezomagnetic structure, are shown in the Figure 4. Figure 4b is the partially enlarged figure of Figure 4a. They have the same geometry: *η* = 2 and *β* = π/6. The phase velocity values of the FGPP structure and piezoelectric structure are almost the same. We can only note that the phase velocity for FGPP structure is little higher than that of the piezoelectric structure in Figure 4b. Moreover, the phase velocity for FGPP structures is higher than that of the piezomagnetic structure. Therefore, the influence of piezoelectric effect is much more significant than that of the piezomagnetic effect, which is similar to other FGPP structures [33]. Figure 5 shows the phase velocity curves of the graded elastic and piezomagnetic structure. We can note that their differences are also very little. The piezomagnetic effect makes the phase velocity decrease only a little.

**Figure 6:**
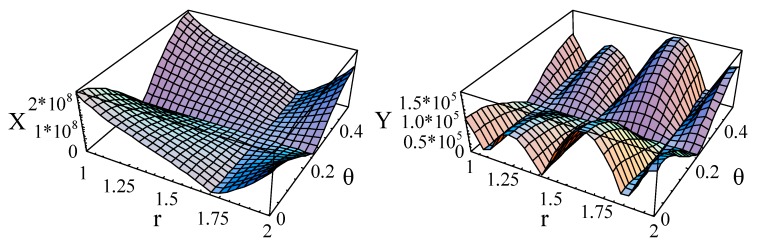
The electric and magnetic potential of the first mode for the linearly cylindrical structure (*η* = 2 and *β* = π/6) at *kd* = 2.01.

**Figure 7:**
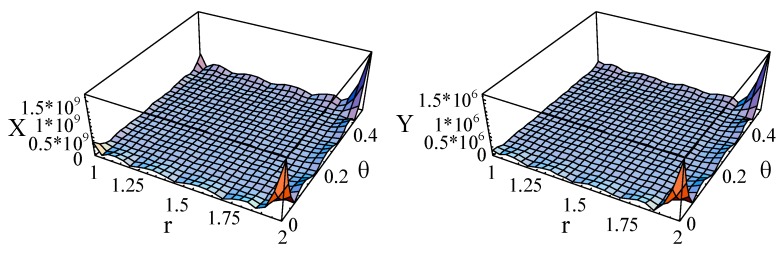
The electric and magnetic potential of the first mode for the linearly cylindrical structure (*η* = 2 and *β* = π/6) at *kd* = 120.01.

Subsequently, the electric and magnetic potential distributions are calculated and illustrated in Figure 6 and Figure 7, at *kd* = 2.01 and *kd* = 120.01, respectively. Here, we just calculated the absolute values of the electric and magnetic potential. Amplitudes of electric potential are much bigger (about 10^3^ times) than that of the magnetic potential, which has a relationship with the electric and magnetic parameters. The phenomenon that the piezoelectric effect is stronger than the piezomagnetic effect is confirmed again. Furthermore, regardless of the amplitude, there are obvious differences between the distribution shapes at small wavenumbers, but they are similar at bigger wavenumbers.

The abovementioned phenomenon can be detailed according to Equation (4) and Table 1 in the reference [34]. We can note from Equation (4) that the piezoelectric parameters and piezomagnetic parameters have the same influence. The piezoelectric and piezomagnetic parameters have a positive relationship with the magneto-electric effect, and the dielectric and magnetic permeability coefficients are negatively related to the magneto-electric effect. Besides, the average absolute values of piezoelectric parameters are about 50 times the piezomagnetic parameters, and the average values of magnetic permeability coefficients are about 5 × 10^6^ times the dielectric coefficients. Hence, the influence of piezomagnetic is very weak, and the amplitude of electric potential is about 10^3^ times of magnetic potential, due to the much higher magnetic permeability coefficients.

### 3.4. The Influence of the Graded Functions

**Figure 8:**
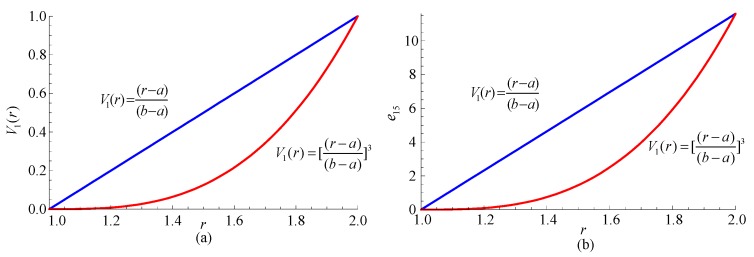
The variation curves of material properties with different graded functions. (**a**) Material volume content for BaTiO_3_; (**b**) e_15_.

**Figure 9:**
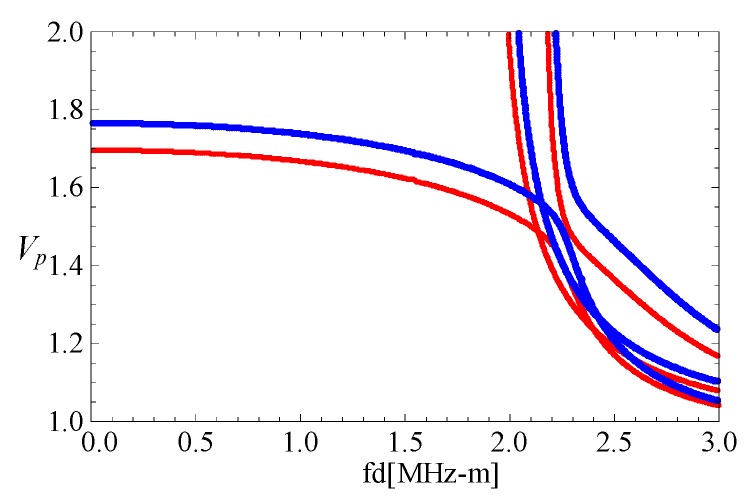
The phase velocity curves with power series functions: red lines—linear function, blue lines—cubic function.

For the FGPP material, the graded function, a significant index in the material design, has significant influence on the material performance. Consequently, it also has remarkable influence on the wave characteristics. To investigate the influence of the graded function, two FGPP cylindrical structures with power series gradient functions are considered, i.e., *V*_1_(*r*) = (*r* − *a*)/*d* and *V*_1_(*r*) = [(*r* − *a*)/*d*]^3^. They have the same geometry: *η* = 2, *β* = π/6. Figure 8 shows variation curves of material properties with different graded functions. Figure 9 illustrates their dispersion curves of the fourth to sixth modes. For a given mode, phase velocities with cubic function are higher than those with a linear function, since it is composed of more CoFe_2_O_4_, and the wave velocity of BaTiO_3_ is lower than that of CoFe_2_O_4_.

**Figure 10:**
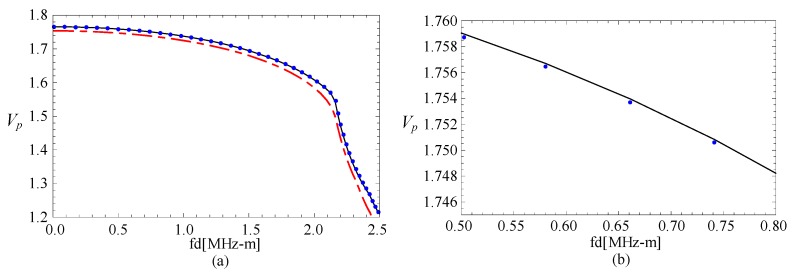
The phase velocity curves with cubic function: the line—FGPP structure, dotted line—piezoelectric structure, dashed line—piezomagnetic structure; (**b**) is the enlarged drawing of (**a**).

Subsequently, the influence of graded function on magneto-electric effect is discussed. Figure 10 shows the corresponding phase velocity curves. Comparing with the Figure 4, we can note that the piezoelectric effect obviously weakens. This is because the volume content for BaTiO_3_ decreases as *n* increases, and the piezoelectric effect mainly results from BaTiO_3_.

### 3.5. The Influence of the Geometric Size

**Figure 11:**
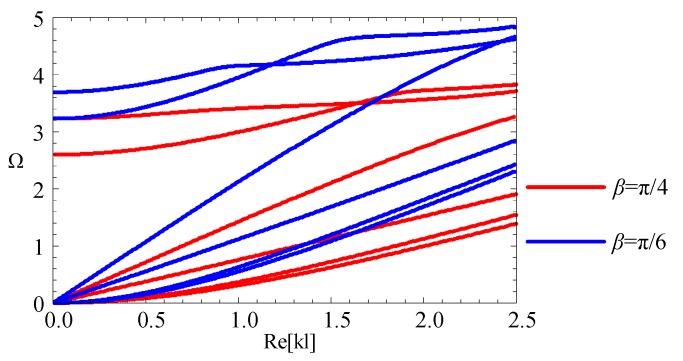
The dispersion curves with different angular measure: red lines—*β* = π/4, blue lines—*β* = π/6.

Control wave characteristics via altering the geometric size is supposed as an efficient approach, such as the cut-off frequency. Accordingly, for the fan-shaped cross-section, the influence of variation in the angular measures and the radius-thickness ratios are investigated. Firstly, two linearly graded cylindrical structures with different angular measures (*β* = π/4, π/6) and *η =* 2 are considered. The dispersion curves are shown in the Figure 11. The first four modes start from 0 frequency. Moreover, for higher modes, the cut-off frequencies increase as angular measures decrease. For a given *η* (for example *η =* 2), as the angular measures decrease, the cross-section area decreases, and the cut-off frequencies increase. Therefore, we hold the view that cut-off frequencies have a negative relationship with the cross-sectional area of the structure.

**Figure 12:**
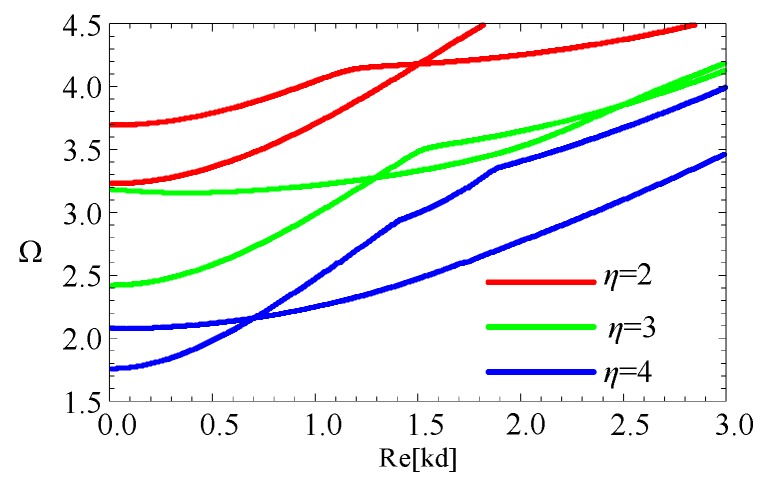
The dispersion curves for fifth and sixth modes with different ratios of the radius-thickness.

Subsequently, to confirm the above view, three linearly graded cylindrical structures, with the radius-thickness ratios (*η* = 2, 3, 4) and *β* = π/6, are also studied. Figure 12 shows their dispersion curves. We can note that the cut-off frequencies decrease as the radius-thickness ratios increase. For the given angular measure (for example *β* = π/6), as the radius-thickness ratios increase, the cross-section area increases, and the cut-off frequency decreases.

**Figure 13:**
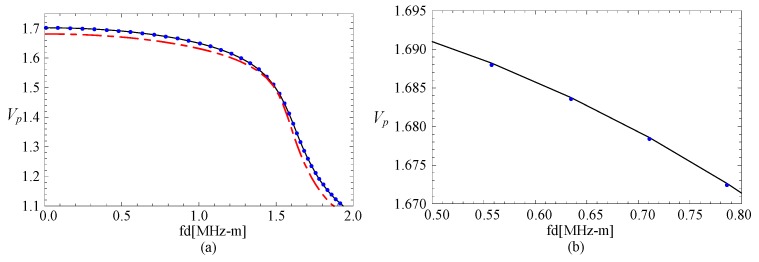
The phase velocity curves for *η* = 3: the line—FGPP structure, dotted line—piezoelectric structure, dashed line—piezomagnetic structure; (**b**) is the enlarged drawing of (**a**).

**Figure 14:**
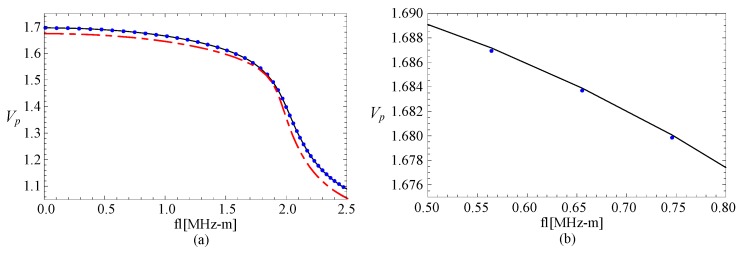
The phase velocity curves for *η* = 2 and *β* = π/4: the line—FGPP structure, dotted line—piezoelectric structure, dashed line—piezomagnetic structure; (**b**) is the enlarged drawing of (**a**).

**Figure 15:**
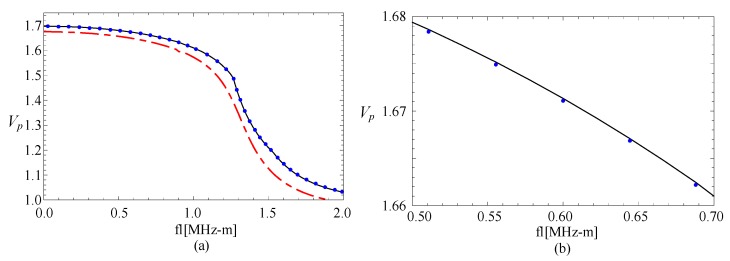
The phase velocity curves for *η* = 2 and *β* = π/8; the line—FGPP structure, dotted line—piezoelectric structure, dashed line—piezomagnetic structure; (**b**) is the enlarged drawing of (**a**).

Then, comparing with 1-D cylindrical structures, there may be some different phenomena because of its complex geometrical shape. Therefore, the influence of the geometric size of the cross-section on the magneto-electric effect is also studied. Figure 13 illustrates phase velocity curves for fan-shaped cylindrical structures with *η* = 3 and *β* = π/6. Comparing with the Figure 4, a phenomenon is found that the piezoelectric effect becomes weak at the transition section (about 1.3–1.6 MHz-m), where the dispersion becomes strong from weakness. Figure 14 and Figure 15 show the corresponding phase velocity curves for the fan-shaped cylindrical structures with *β* = π/4 and *β* = π/8, respectively. It can be seen from these two figures that, for the fan-shaped cylindrical structures with *β* = π/4, the influence of piezoelectric effect also becomes very weak at the transition section (about 1.6–2.1 MHz-m), where the dispersion becomes strong from weakness. In summary, the magneto-electric effect could be adjusted via altering the geometric size.

### 3.6. Waves at High Frequencies

**Figure 16:**
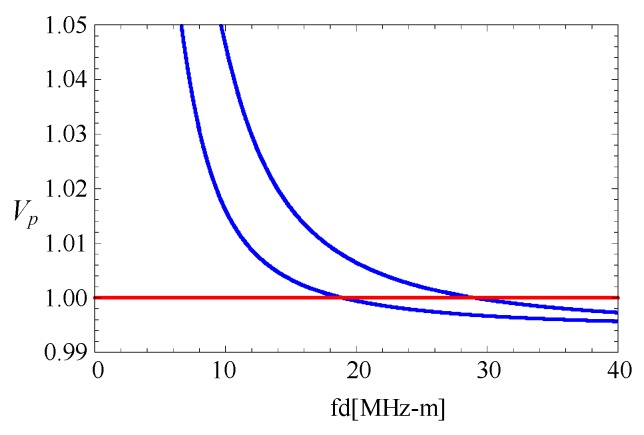
Phase velocity curves for the fifth and sixth mode with *β* = π/6 and *η* = 2.

For the 1-D cylindrical structures [25], the phase velocity values of higher modes always increase as the frequency decreases, and are close to the shear velocity *c_s_*. However, the case for 2-D fan-shaped cylindrical structures is quite different. A homogeneous (*V*_1_(*r*) = 1) fan-shaped cylindrical structure with *η* = 2 and *α* = π/6 is taken into account. The phase velocity curves for the fifth and sixth mode are shown in Figure 16. The velocity values at high frequencies approximatively approach a value which is below *c_s_*. This is because wave motions at high frequencies mainly concentrate near corners of the fan-shaped cylindrical structures, while the other places remain almost motionless (see in the Section 3.8). The velocity for waves propagating near the edges at high frequencies is lower than that for waves propagating at a surface.

**Figure 17:**
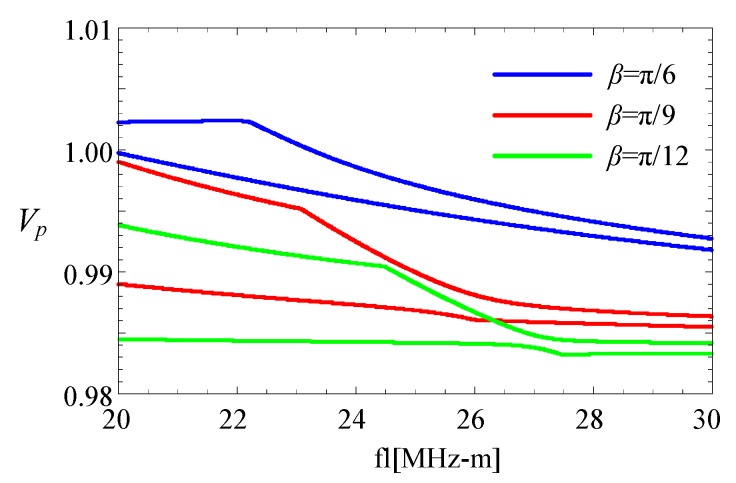
Phase velocity curves for the fifth and six mode at *η* = 2.

Subsequently, two linear FGPP fan-shaped cylindrical structures with different angular measures are considered. Figure 17 shows the corresponding phase velocity curves at high frequencies. Phase velocity values of the fan-shaped cylindrical structures, with *β* = π/6, are higher than that of structure with *β* = π/9 and *β* = π/12, i.e., phase velocity values at high frequencies decrease as with the decrease of *β*. This feature is similar to that of wedge waves.

### 3.7. The Stress, Electric, and Magnetic Displacement Distribution

**Figure 18:**
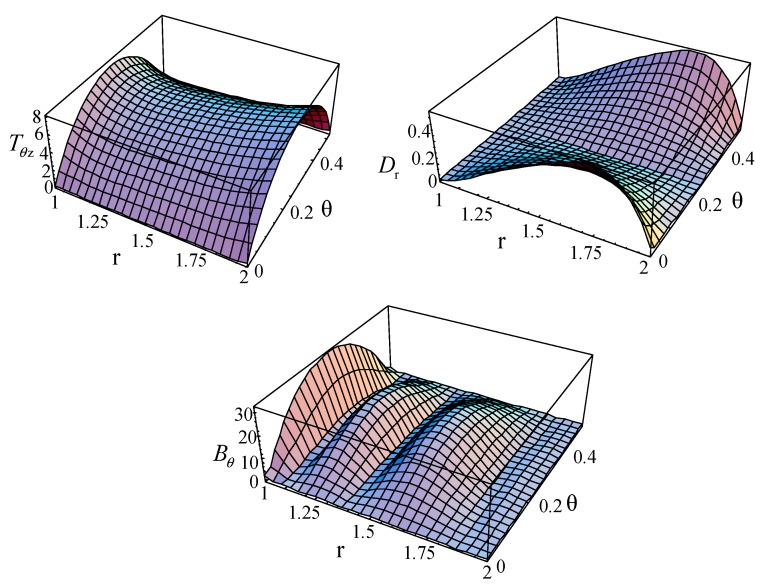
The stress, electric, and magnetic displacement of the first mode for a linearly FGPP cylindrical structure (*η* = 2 and *β* = π/6) at *kd* = 2.01.

The stress, electric displacement, and magnetic displacement of the first mode for a linear FGPP fan-shaped cylindrical structure (*η* = 2 and *β* = π/6) at *kd* = 2.01 are calculated and illustrated in Figure 18. Here, to save the space, only three representative figures are illustrated. It can be easily seen that the initial conditions are satisfied extremely well, i.e., $T_{rr}=T_{r\theta }=T_{rz}=D_{r}=B_{r}=0$ at *r* = *a* and *r* = *b*; and $T_{\theta \theta }=T_{r\theta }=T_{\theta z}=D_{\theta }=B_{\theta }=0$ at *θ* = 0 and *θ* = *β*.

### 3.8. The Poynting Vector

It is well known that the Poynting vector denotes the power flow density, which can be calculated by

(10a) Pr=Re[0.5iω(Trr×ur*+Trθ×uθ*+Trz×uz*+Dr×ϕ*+Br×Ψ*)],

(11b) Pθ=Re[0.5iω(Trθ×ur*+Tθθ×uθ*+Tθz×uz*+Dθ×ϕ*+Bθ×Ψ*)],

(10c) Pz=Re[0.5iω(Trz×ur*+Tθz×uθ*+Tzz×uz*+Dz×ϕ*+Bz×Ψ*)],

where superscript * denotes the complex conjugation.

**Figure 19:**
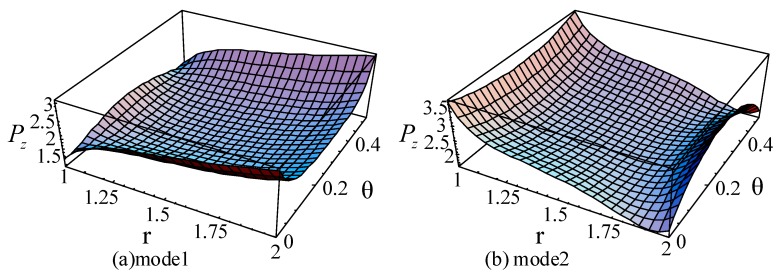
The Poynting vector of the first two modes for the linearly cylindrical structure (*η* = 2 and *β* = π/6) at *kd* = 2.01. (**a**): mode 1; (**b**): mode 2.

**Figure 20:**
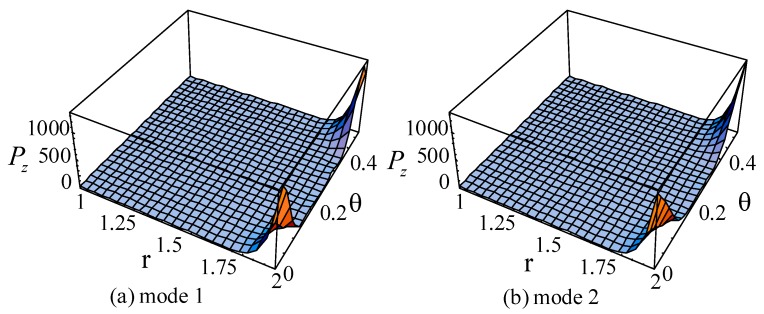
The Poynting vector of the first two modes for the linearly cylindrical structure (*η* = 2 and *β* = π/6) at *kd* = 120.01. (**a**): mode 1; (**b**): mode 2.

**Figure 21:**
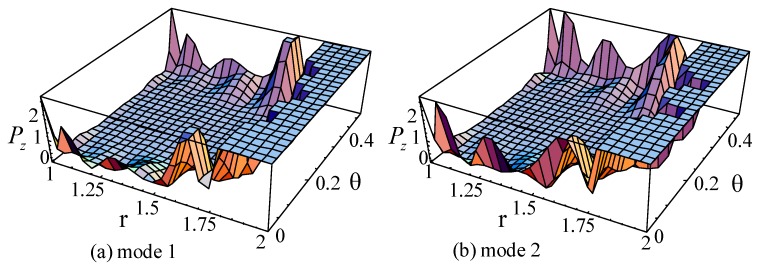
The partial enlarged drawings of the Figure 20. (**a**): mode 1; (**b**): mode 2.

For the guided waves propagating in the axial direction, the Poynting vectors in the radial and circumference directions are 0. Figure 19, Figure 20 and Figure 21 illustrate the Poynting vector distributions of the first two modes for the linear FGPP cylindrical structure (*η* = 2 and *β* = π/6), at *kd* = 2.01 and *kd* = 120.01, respectively. The detailed distributions at *kd* = 120.01 in the Figure 20 cannot be clearly seen. Hence, the partial enlarged drawings are made, as shown in the Figure 21. They seem to be discontinuous, owing to the existence of platforms. However, they are continuous, in fact, and truncation of the larger values leads to platforms. Furthermore, for the big wavenumber case, the Poynting vector distributions are mainly concentrated near the outer side with more BaTiO_3_. This is because elasticity modulus of BaTiO_3_ is smaller than that of CoFe_2_O_4_. Accordingly, the stiffness of BaTiO_3_ is smaller, the displacement is bigger, and the energy mainly transmits in this region. Moreover, the Poynting vector distributions are concentrated near the boundaries, especially near the corner.

## 4. Conclusions

According to the 3D linearly magneto-electric-elastic theory, waves in FGPP fan-shaped cylindrical structures are studied via exploiting the double Legendre orthogonal polynomial method. The magneto-electric effect is detailed. Based on the above numerical results, the following conclusions can be obtained:

(1) If the radius-thickness ratio is bigger than 1000, fan-shaped cylindrical structures could be treated as a rectangular bar.
(2) The variation in geometric size of the cross-section has remarkable influence on wave characteristics. The cut-off frequencies have a negative relationship with the cross-section area of the fan-shaped cylindrical structures.
(3) For the FGPP fan-shaped cylindrical structures, the magneto-electric effect could be adjusted via altering the geometric size.
(4) The phase velocities for higher modes at high frequencies approximatively approach a value which is below the shear velocity, and they also increase with the increase of *β*.
(5) For the big wavenumber case, the Poynting vector distributions are concentrated in the region with more material of smaller elasticity modulus, and the Poynting vector distributions are also concentrated near the boundaries, especially near the corner.

## Figures and Tables

**Table 1:** Phase velocities of the first three modes at *kd* = 1.01.

M,J	Mode1	Mode2	Mode3
**4,4**	1054.86	1221.42	2564.77
**4,5**	1054.80	1221.34	2563.99
**4,6**	1054.79	1221.34	2563.12
**5,4**	1054.24	1220.87	2562.70
**5,5**	1054.19	1220.79	2561.84
**5,6**	1054.19	1220.79	2561.10
**6,4**	1054.27	1220.83	2560.59
**6,5**	1054.23	1220.75	2559.78
**6,6**	1054.22	1220.75	2559.17

unit: m/s.

## Appendix A

Equation (7e) can be transformed as

(A1) $$p_{m,j}^{5}=-{\left({}^{l}A_{55}^{n,p,m,j}\right)}^{-1}\left({}^{l}A_{51}^{n,p,m,j}p_{m,j}^{1}+{}^{l}A_{52}^{n,p,m,j}p_{m,j}^{2}+{}^{l}A_{53}^{n,p,m,j}p_{m,j}^{3}+{}^{l}A_{54}^{n,p,m,j}p_{m,j}^{4}\right).$$

Subsequently, substituting Equation (A1) into Equation (7d), we can obtain the following equation.

(A2) $$\begin{array}{l} p_{m,j}^{4}={}^{l}IA^{n,p,m,j}\{[{}^{l}A_{41}^{n,p,m,j}-{}^{l}AA^{n,p,m,j}\cdot {}^{l}A_{51}^{n,p,m,j}]p_{m,j}^{1}\\ +\left[{}^{l}A_{42}^{n,p,m,j}-{}^{l}AA^{n,p,m,j}\cdot {}^{l}A_{52}^{n,p,m,j}\right]p_{m,j}^{2}+\left[{}^{l}A_{43}^{n,p,m,j}-{}^{l}AA^{n,p,m,j}\cdot {}^{l}A_{53}^{n,p,m,j}\right]p_{m,j}^{3}\} \end{array}$$

where ${}^{l}AA^{n,p,m,j}={}^{l}A_{45}^{n,p,m,j}{\left({}^{l}A_{55}^{n,p,m,j}\right)}^{-1}$ and ${}^{l}IA^{n,p,m,j}={\left[{}^{l}A_{45}^{n,p,m,j}{\left({}^{l}A_{55}^{n,p,m,j}\right)}^{-1}\cdot {}^{l}A_{54}^{n,p,m,j}-{}^{l}A_{44}^{n,p,m,j}\right]}^{-1}$.

Then, substituting Equation (A2) into Equation (A1)

(A3) $$\begin{array}{l} p_{m,j}^{5}=-{\left({}^{l}A_{55}^{n,p,m,j}\right)}^{-1}\{{}^{l}A_{51}^{n,p,m,j}p_{m,j}^{1}+{}^{l}A_{52}^{n,p,m,j}p_{m,j}^{2}+{}^{l}A_{53}^{n,p,m,j}p_{m,j}^{3}\\ +{}^{l}A_{54}^{n,p,m,j}\cdot {}^{l}IA^{j,m}[\left({}^{l}A_{41}^{n,p,m,j}-{}^{l}AA^{n,p,m,j}\cdot {}^{l}A_{51}^{n,p,m,j}\right)p_{m,j}^{1}\\ +\left({}^{l}A_{42}^{n,p,m,j}-{}^{l}AA^{n,p,m,j}\cdot {}^{l}A_{52}^{n,p,m,j}\right)p_{m,j}^{2}+\left({}^{l}A_{43}^{n,p,m,j}-{}^{l}AA^{n,p,m,j}\cdot {}^{l}A_{53}^{n,p,m,j}\right)p_{m,j}^{3}]\} \end{array}.$$

Finally, substituting Equations (A2) and (A3) into Equations (7a), (7b), and (7c), Equation (8) can be obtained.

## References

[1] Qin G., Lu C., Zhang X. (2018). Electric Current Dependent Fracture in GaN Piezoelectric Semiconductor Ceramics. Materials.

[2] Ishaq S., Kanwal F., Atiq S., Moussa M., Azhar U., Imran M., Losic D. (2018). Advancing Dielectric and Ferroelectric Properties of Piezoelectric Polymers by Combining Graphene and Ferroelectric Ceramic Additives for Energy Storage Applications. Materials.

[3] Drahus M.D., Jakes P., Erdem E. (2011). Manganese-doped (1 − *x*) BiScO_3_ − *x* PbTiO_3_, high-temperature ferroelectrics: Defect structure and mechanism of enhanced electric resistivity. Phys. Rev. B.

[4] Kostsov E.G. (2006). Ferroelectric-based electrostatic micromotors with nanometer gaps. IEEE Trans. Ultrason. Ferroelectr..

[5] Jones K.A., Chow T.P., Wraback M., Sitar Z., Shahedipour F., Udwary K.G., Tompa S. (2015). AlGaN devices and growth of device structures. J. Mater. Sci..

[6] Erdem E., Eichel R.A., Kungl H., Hoffmann M.J., Ozarowski A., Tol H., Brunel L.C. (2007). Local symmetry-reduction in tetragonal (La,Fe)-codoped Pb[Zr0.4Ti0.6]O_3_ piezoelectric ceramics. Phys. Scr..

[7] Bishay P.L., Sladek J., Sladek V., Atluri S.N. (2012). Analysis of Functionally Graded Piezoelectric-piezomagnetic Composites Using Hybrid/Mixed Finite Elements and Node-Wise Material Properties. CMC-Comput. Mater. Contin..

[8] Afonin S.M. (2010). Static and dynamic characteristics of multilayered electromagnetoelastic transducer of nano- and micrometric movements. Sov. J. Comput. Syst. Sci..

[9] Chen L., Li P., Wen Y. (2012). The magnetostrictive material effects on magnetic field sensitivity for magnetoelectric sensor. J. Appl. Phys..

[10] Cao X., Shi J., Jin F. (2012). Lamb wave propagation in the functionally graded piezoelectric–piezomagnetic material plate. Acta Mech..

[11] Singh B.M., Rokne J. (2013). Propagation of SH waves in layered functionally gradient piezoelectric–piezomagnetic structures. Philos. Mag..

[12] Yu J., Wu B. (2009). Circumferential wave in piezoelectric-piezomagnetic functionally graded cylindrical curved plates. Eur. J. Mech..

[13] Li P., Jin F., Qian Z. (2013). Propagation of the Bleustein–Gulyaev waves in a functionally graded transversely isotropic electro-magneto-elastic half-space. Eur. J. Mech. A Solids.

[14] Li L., Wei P.J. (2014). Surface Wave Speed of Functionally Graded Magneto-Electro-Elastic Materials with Initial Stresses. J. Theor. Appl. Mech..

[15] Xue C.X., Pan E. (2013). On the longitudinal wave along a functionally graded piezoelectric-piezomagnetic rod. Int. J. Eng. Sci..

[16] Arefi M. (2016). Analysis of wave in a functionally graded piezoelectric-piezomagnetic nano-rod using nonlocal elasticity model subjected to electric and magnetic potentials. Acta Mech..

[17] Narendar S. (2016). Wave dispersion in functionally graded piezoelectric-piezomagnetic nonlocal rod. Aerosp. Sci. Technol..

[18] Shen X., Ren D., Cao X. (2018). Cut-off frequencies of circumferential horizontal shear waves in various functionally graded cylinder shells. Ultrasonics.

[19] Shodja H.M., Eskandari S., Eskandari M. (2016). Shear horizontal surface acoustic waves in functionally graded magneto-electro-elastic half-space. J. Eng. Math..

[20] Ezzin H., Ben A.M., Ghozlen M.H. (2016). Love waves propagation in a transversely isotropic piezoelectric layer on a piezomagnetic half-space. Ultrasonics.

[21] Ezzin H., Mkaoir M., Amor M.B. (2017). Rayleigh wave behavior in functionally graded magneto-electro-elastic material. Superlattices Microstruct..

[22] Ezzin H., Ben A.M., Ghozlen M.H. (2017). Lamb waves propagation in layered piezoelectric/piezomagnetic plates. Ultrasonics.

[23] Singhal A., Sahu S.A. (2018). Chaudhary, S. Approximation of surface wave frequency in piezo-composite structure. Compos. Part B Eng..

[24] Zhang B., Yu J., Shah A.A., Yang X. (2017). Wave propagation in functionally graded piezoelectric-piezomagnetic rectangular bars. Sci. Eng. Compos. Mater..

[25] Zhou Y., Chen W., Lü C. (2012). Elastic waves in multiferroic cylinders of sectorial cross-section. Compos. Part B.

[26] Puzyrev V. (2010). Elastic waves in piezoceramic cylinders of sector cross-section. Int. J. Solids Struct..

[27] Puzyrev V., Storozhev V. (2011). Wave propagation in axially polarized piezoelectric hollow cylinders of sector cross section. J. Sound Vib..

[28] Storozhev V.I. (2013). Propagation of Electroelastic Waves in Multilayer Piezoelectric Cylinders with a Sector Notch. Int. Appl. Mech..

[29] Zhang B., Yu J.G., Zhang X.M., Ming P.M. (2018). Complex guided waves in functionally graded piezoelectric cylindrical structures with sectorial cross-section. Appl. Math. Model..

[30] Zuo P., Zhou Y., Fan Z. (2016). Numerical studies of nonlinearly ultrasonic guided waves in uniform waveguides with arbitrary cross sections. Aip Adv..

[31] Fan Z., Lowe M.J., Castaings M. (2008). Torsional waves propagation along a waveguide of arbitrary cross section immersed in a perfect fluid. J. Acoust. Soc. Am..

[32] Zhang B., Yu J.G., Zhang X.M. (2017). Guided wave propagation in cylindrical structures with sector cross-sections. Arch. Appl. Mech..

[33] Yu J., Ma Q., Su S. (2008). Wave propagation in non-homogeneous magneto-electro-elastic hollow cylinders. Ultrasonics.

[34] Srinivas S., Li J.Y. (2006). The effective magneto-electro-elastic moduli of matrix-based multiferroic composites. J. Appl. Phys..

[35] Liu Y., Han Q., Huang H., Li C., Liu X., Wu B. (2015). Computation of dispersion relations of functionally graded rectangular bars. Compos. Struct..

